# The limitation of genetic testing in diagnosing patients suspected for congenital platelet defects

**DOI:** 10.1002/ajh.25667

**Published:** 2019-11-13

**Authors:** Maaike W. Blaauwgeers, Ivar van Asten, Marieke J.H.A. Kruip, Erik A.M. Beckers, Michiel Coppens, Jeroen Eikenboom, Karin P.M. van Galen, Albert Huisman, Suzanne J.A. Korporaal, Hans Kristian Ploos van Amstel, Rienk Y.J. Tamminga, Rolf T. Urbanus, Roger E.G. Schutgens

**Affiliations:** ^1^ Van Creveldkliniek University Medical Center Utrecht, University Utrecht Utrecht The Netherlands; ^2^ Van Creveld Laboratory University Medical Center Utrecht, University Utrecht Utrecht The Netherlands; ^3^ Center for Circulatory Health, Department of Clinical Chemistry and Haematology University Medical Center Utrecht, University Utrecht Utrecht The Netherlands; ^4^ Department of Haematology Erasmus University Medical Center Rotterdam The Netherlands; ^5^ Department of Hematology Maastricht University Medical Center Maastricht The Netherlands; ^6^ Department of Vascular Medicine, Amsterdam Cardiovascular Sciences Amsterdam University Medical Center, location AMC Amsterdam The Netherlands; ^7^ Department of Internal Medicine, division of Thrombosis and Haemostasis Leiden University Medical Center Leiden The Netherlands; ^8^ Laboratory of Experimental Cardiology University Medical Center Utrecht, University Utrecht Utrecht The Netherlands; ^9^ Department of Medical Genetics University Medical Center Utrecht, University Utrecht Utrecht The Netherlands; ^10^ Department of Pediatric Hematology Beatrix Children's Hospital, University Medical Center Groningen Groningen The Netherlands

1

To the Editor:

Congenital platelet defects (CPD) are rare disorders of primary hemostasis caused by congenital defects in platelet production or function. Identification of CPDs is challenging due to the lack of awareness resulting in late or missing referrals, the lack of diagnostic criteria, absence or limitations of laboratory tests and poor standardization of the available tests.^1^ However, an accurate diagnosis is important for proper counseling and management of patients and to avoid ineffective and potentially harmful treatments due to misdiagnosis, like idiopathic thrombocytopenic purpura (ITP).

DNA‐based analysis has become increasingly important for diagnosing CPDs.^2^ Genetic analysis can be useful to confirm a suspected phenotypic diagnosis, and to identify patients with an increased risk for associated pathologies, such as myelofibrosis (*NBEAL2*), renal insufficiency (*MYH9*) and hematological malignancies (*RUNX1*). The International Society for Thrombosis and Hemostasis (ISTH) currently recommends to perform genetic analysis as a third‐line investigation, that is, after extensive phenotyping and functional analyses have confirmed the presence of a platelet disorder.^3^ Recent studies on the efficacy of genetic testing in selected patients with platelet disorders have suggested that genetic analysis could be moved “upward” in the diagnostic approach in order to simplify and hasten the diagnosis of CPDs.^4^, ^5^, ^6^ However, it remains unclear whether genetic analysis should be performed as a first‐line investigation, alongside initial functional analysis of platelet function in unselected patients in whom a congenital platelet disorder is suspected.

In the Thrombocytopathy in the Netherlands (TiN) study, we assessed the diagnostic value of genetic analysis performed in parallel with routine laboratory tests in a prospective cohort of patients suspected of having a CPD. Three categories of patients were included in the study: (a) patients suspected of having a CPD based on previous abnormal platelet counts, light transmission aggregometry (LTA) results or platelet ADP content without a molecular diagnosis (n = 96) (b) patients suspected of having a CPD based on a predominantly mucocutaneous bleeding tendency compatible with a CPD, in whom other known causes of bleeding were excluded and in whom previous LTA results were normal (n = 39), and (c) patients suspected of having a CPD based on a predominantly mucocutaneous bleeding tendency compatible with a CPD, in whom other known causes of bleeding were excluded, newly referred for platelet function testing (n = 21). Laboratory tests were performed for platelet count, aggregation response to four agonists, nucleotide content, surface receptor expression with flow cytometry and whole‐exome sequencing (WES) with a selected 76 gene panel (Table S1). A CPD was diagnosed when an abnormal platelet count or function was found on at least two separate occasions, of which one was in our diagnostic laboratory. A possible CPD was diagnosed when an abnormal platelet function was found once in our diagnostic laboratory, or when abnormal platelet function test results were inconsistent with previous findings. In line with the American College of Medical Genetics guidelines, a genetic variant was stated to be causal when a (likely) pathogenic variant (class 4 or 5, respectively)^7^ was identified in one or more of the selected genes that corresponded to the platelet phenotype.

In patients with previously abnormal laboratory results, a CPD was confirmed in 61 of 96 (64%) patients, and a possible CPD was diagnosed in four of 96 (4%) patients. Eight of 96 (8%) patients received a molecular diagnosis, and in 11 of 96 (11%) patients a variant of unknown significance was identified (Table 1). In patients with previously normal LTA results and in newly referred patients, a possible CPD was diagnosed in 10 of 39 (26%) and six of 21 (29%) patients, respectively. No causal genetic variants were identified in these patients.

**Table 1:** Results of laboratory and genetic testing per patient category

Patient category	N	CPD	Possible CPD	Molecular diagnosis	VUS
Previously abnormal laboratory tests^a^	96	61 (64)	4 (4)	8 (8)	11 (11)
Previously normal LTA results^b^	39	0 (0)	10 (26)	0 (0)	1 (3)
Newly referred^c^	21	0 (0)	6 (29)	0 (0)	1 (5)

Note: Data are presented in number of patients (%).

Abbreviations: CPD, congenital platelet defect; LTA, light transmission aggregometry; VUS, variants of uncertain significance.

a Patients suspected for a CPD based on previous abnormal platelet counts, LTA results or platelet ADP content without a molecular diagnosis.

b Patients suspected for a CPD based on a predominantly mucocutaneous bleeding tendency compatible with a CPD, in whom other known causes of bleeding were excluded and in whom previous LTA results were normal.

c Patients suspected for a CPD based on a predominantly mucocutaneous bleeding tendency compatible with a CPD, in whom other known causes of bleeding were excluded, newly referred for platelet function testing.

We included several subgroups of patients suspected of having a CPD to properly assess when genetic analysis should be performed in the diagnostic procedure. Our study shows that the diagnostic yield of genetic analysis is limited in patients suspected for a CPD, since only 5% (8/156) of patients received a molecular diagnosis. This is in contrast to the diagnostic rate of 47.8% for platelet count defects, and 26.1% for platelet function defects reported in a recent study. There, 2396 patients with bleeding, thrombotic, and platelet disorders (BTPD) were screened with a panel of 96 BTPD‐associated genes, in which the number of platelet associated genes was similar to our gene panel.^8^ However, their diagnostic rate included variants of unknown significance, resulting in an overestimation. Leaving out variants of unknown significance strongly reduced the diagnostic rate. The differences between their and our study are also related to patient‐selection. Our study reflects the real‐life population of patients suspected for a CPD referred to outpatient clinics of hemophilia treatment centers Their study included patients with a previously ascertained pathogenic variant, or patients with phenotypes strongly indicative of a particular disorder on the basis of laboratory abnormalities, with a high likelihood of having an inherited BTPD. In patients with either normal laboratory assays or assays not diagnostic of an established disorder, they reported a diagnostic rate of only 3.2%. Studies performed in the Iberian Peninsula, with a gene panel similar to ours, reported the identification of a molecular defect in 40%^9^ and 68%^5^ of patients with (suspected) CPDs. Their studies included large numbers of patients with Glanzmann thrombasthenia, Bernard‐Soulier syndrome and MYH9‐related disorders. Therefore, their study population does not reflect clinical practice and is not comparable to ours. A study performed in a pediatric population reported a positive molecular diagnosis in 23.8%.^10^ Their cohort included a relatively large number of patients with thrombocytopenia (67% vs 22% in our cohort) and genetic testing was not performed as a first‐line investigation.

It is possible that limitations of WES have led to an underestimation of the number of patients with an identified genetic variant. First, large insertions and deletions might be missed. Second, regulatory and non‐coding regions of the genome were not examined, and these regions might harbor variants essential for controlling transcriptional regulation or splicing. Third, by using a selected gene panel we might have missed pathogenic variants in genes not included in the panel. Finally, we cannot exclude that an additive effect of multiple genetic variants that have escaped our selection, might underlie the CPD in individual patients.

In conclusion, genetic testing with a selected gene panel has limited diagnostic yield in patients suspected for a CPD and should only be performed in patients in whom a platelet number or function defect is confirmed.

## CONFLICT OF INTEREST

The authors state that they have no conflict of interest.

## Supporting information


**Supplementary Table 1** Genes included in the WES gene panel for molecular screening of primary hemostatic disorders

## References

[1] Dovlatova N . Current status and future prospects for platelet function testing in the diagnosis of inherited bleeding disorders. Br J Haematol. 2015;170(2):150‐161. 25920378 10.1111/bjh.13405

[2] Lentaigne C , Freson K , Laffan MA , Turro E , Ouwehand WH . Inherited platelet disorders: towards DNA‐based diagnosis. Blood. 2016;127:2814‐2823. 27095789 10.1182/blood-2016-03-378588 PMC4972611

[3] Gresele P . Diagnosis of inherited platelet function disorders: guidance from the SSC of the ISTH. J Thromb Haemostasis. 2015;13(2):314‐322. 25403439 10.1111/jth.12792

[4] Daly ME , Leo VC , Lowe GC , Watson SP , Morgan NV . What is the role of genetic testing in the investigation of patients with suspected platelet function disorders? Br J Haematol. 2014;165(2):193‐203. 24479992 10.1111/bjh.12751

[5] Bastida JM , Lozano ML , Benito R , et al. Introducing high‐throughput sequencing into mainstream genetic diagnosis practice in inherited platelet disorders. Haematologica. 2018;103(1):148‐162. 28983057 10.3324/haematol.2017.171132 PMC5777202

[6] Bariana TK , Ouwehand WH , Guerrero JA , Gomez K , the BRIDGE Bleeding, Thrombotic and Platelet Disorders and ThromboGenomics Consortia . Dawning of the age of genomics for platelet granule disorders: improving insight, diagnosis and management. Br J Haematol. 2017;176(5):705‐720. 27984638 10.1111/bjh.14471

[7] Richards S , Aziz N , Bale S , et al. Standards and guidelines for the interpretation of sequence variants: a joint consensus recommendation of the American College of Medical Genetics and Genomics and the Association for Molecular Pathology. Genet Med. 2015;17(5):405‐424. 25741868 10.1038/gim.2015.30 PMC4544753

[8] Downes K , Megy K , Duarte D , et al. Diagnostic high‐throughput sequencing of 2,396 patients with bleeding, thrombotic and platelet disorders. Blood. 2019 blood.2018891192. 10.1182/blood.2018891192. [Epub ahead of print] PMC6993014 31064749

[9] Sanchez‐Guiu I , Anton AI , Padilla J , et al. Functional and molecular characterization of inherited platelet disorders in the Iberian Peninsula: results from a collaborative study. Orphanet J Rare Dis. 2014;9:213. 25539746 10.1186/s13023-014-0213-6 PMC4302577

[10] Romasko EJ , Devkota B , Biswas S , et al. Utility and limitations of exome sequencing in the molecular diagnosis of pediatric inherited platelet disorders. Am J Hematol. 2018;93(1):8‐16. 28960434 10.1002/ajh.24917 PMC6366456

