# Supplementary material for: The limitation of genetic testing in diagnosing patients suspected for congenital platelet defects
Source: Am J Hematol. 2019 Nov 13;95(1):E26–8. doi: 10.1002/ajh.25667 (PMC6916199; doi:10.1002/ajh.25667)
Supplement: Supplementary file 1 — Supplementary Table 1 Genes included in the WES gene panel for molecular screening of primary hemostatic disorders [file AJH-95-E26-s001.docx]

Supplementary Table 1. Genes included in the WES gene panel for molecular screening of primary hemostatic disorders

| **Target protein** | **Gene** | **Description** | **Gene** | **Description** |
| --- | --- | --- | --- | --- |
| Platelet agonist receptors | *ADRA2A* | G-protein coupled receptors | *GP9* | Bernard Soulier syndrome |
|  | *ADRA2B* | G-protein coupled receptors | *ITGA2* | Bleeding disorder, platelet type 9 |
|  | *CD36* | Bleeding disorder, platelet type 10 | *ITGA2B* | Glanzmann thrombasthenia |
|  | *F2R* | G-protein coupled receptors | *ITGB1* | Bleeding disorder, platelet type 9 |
|  | *F2R13* | G-protein coupled receptors | *ITGB3* | Glanzmann thrombasthenia |
|  | *GP1BA* | Bernard Soulier syndrome | *P2RY12* | Bleeding disorder, platelet type 8 |
|  | *GP1BB* | Bernard Soulier syndrome | *TBXA2R* | Bleeding disorder, platelet type 13 |
|  | *GP6* | Bleeding disorder, platelet type 11 |  |  |
| Platelet granules | *AP3B1* | Hermansky-Pudlak syndrome 2 | *LYST* | Chediak-Higashi syndrome |
|  | *BLOC1S3* | Hermansky-Pudlak syndrome 8 | *MLPH* | Griscelli syndrome |
|  | *BLOC1S6* | Hermansky-Pudlak syndrome 9 | *MYO5A* | Griscelli syndrome |
|  | *DTNBP1* | Hermansky-Pudlak syndrome 7 | *NBEAL2* | Gray platelet syndrome |
|  | *HPS1* | Hermansky-Pudlak syndrome 1 | *PLAU* | Quebec platelet disorder |
|  | *HPS3* | Hermansky-Pudlak syndrome 3 | *RAB27A* | Griscelli syndrome |
|  | *HPS4* | Hermansky-Pudlak syndrome 4 | *VPS33B* | ARC syndrome |
|  | *HPS5* | Hermansky-Pudlak syndrome 5 | *VIPAS39A* | ARC syndrome |
|  | *HPS6* | Hermansky-Pudlak syndrome 6 |  |  |
| Signal transduction | *PLA2G4A* | Phospholipase A2 deficiency | *RGS2* | G-protein signaling |
|  | *PTGS1* | Bleeding disorder, platelet type 12 | *TBXAS1* | Bleeding disorder, platelet type 14 |
|  | *RASGRP2* | Bleeding disorder, platelet type 18 |  |  |
| Transcription factors | *CYCS* | Thrombocytopenia 4 | *HOXA11* | CTRUS syndrome |
|  | *ETV6* | Thrombocytopenia 5 | *MECOM* | CTRUS syndrome |
|  | *FLI1* | Bleeding disorder, platelet type 21 | *RBM8A* | TAR syndrome |
|  | *GATA1* | GATA1-related disorder | *RUNX1* | FPD/AML |
|  | *GFI1B* | Bleeding disorder, platelet type 17 | *STIM1* | Stormorken syndrome |
| Cytoskeletal and structural proteins | *ABCG5* | Sitosterolemia | *FYB* | CARST syndrome |
|  | *ABCG8* | Sitosterolemia | *MASTL* | Thrombocytopenia 2 |
|  | *ACTN1* | Bleeding disorder, platelet type 15 | *MYH9* | MYH9-related disorders |
|  | *ANKRD26* | Thrombocytopenia 2 | *PRKACG* | Bleeding disorder, platelet type 19 |
|  | *CDC42* | Takenouchi-Kosaki syndrome | *TUBB1* | TUBB1-related macrothrombocytopenia |
|  | *FERMT3* | Leukocyte adhesion deficiency III | *WAS* | Wiskott-Aldrich syndrome |
|  | *FLNA* | Filaminopathy |  |  |
| Procoagulant disorders | *ANO6* | Scott syndrome |  |  |
| Collagen disorders | *COL1A1* | Ehlers-Danlos syndrome | *COL5A1* | Ehlers-Danlos syndrome |
|  | *COL3A1* | Ehlers-Danlos syndrome | *COL5A2* | Ehlers-Danlos syndrome |
| Blood vessel abnormalities | *ACVRL1* | Hereditary telangiectasia | *ENG* | Hereditary telangiectasia |
| Fibrinogen disorders | *FGA* | Dys/hypo/afibrinogenemia | *FGG* | Dys/hypo/afibrinogenemia |
|  | *FGB* | Dys/hypo/afibrinogenemia |  |  |
| Other | *GBA* | Gaucher disease | *SLFN14* | Bleeding disorder, platelet type 20 |
|  | *GNE* | GNE myopathy | *THPO* | Thrombocytemia 1 |
|  | *MPL* | CAMT syndrome | *VWF* | von Willebrand disease |

ARC: arthrogryposis, renal dysfunction and cholestasis; CAMT: congenital amegakaryocytic thrombocytopenia; CARST: congenital autosomal recessive small-platelet thrombocytopenia; CTRUS: congenital thrombocytopenia with radioulnar synostosis; FPD/AML: familial platelet disorder with propensity to acute myelogenous leukemia; TAR: thrombocytopenia and absent radius;
